# Clinical Outcomes and Complications of Endoscopic Biliary Stenting for Malignant Distal Biliary Obstruction in Pancreatic Cancer: A Systematic Review

**DOI:** 10.3390/jcm15083126

**Published:** 2026-04-20

**Authors:** Nurken Abdiyev, Nurlan Jaxymbayev, Melis Maira, Medet Rakhmetov, Almas Ismailov, Abdykadyrov Mazhit, Yerlan Abdirashev, Berik Dzhumabekov

**Affiliations:** 1 Private Hospital International Almaty, Almaty 050000, Kazakhstan; abdievnm@gmail.com; 2 Kyrgyz State Medical Institute of Retraining and Advanced Training Named After S.B. Daniyarov, Bishkek 720040, Kyrgyzstan; ndzhaksymbaev@bk.ru; 3 Korday District Hospital, Korday 080400, Kazakhstan; 4 Department of Medicine, Al-Farabi Kazakh National University, Almaty 050000, Kazakhstan; mairaimangali210@gmail.com; 5 Medical Center Hospital of the President’s Affairs Administration of the Republic of Kazakhstan, Astana 010000, Kazakhstan; m.m.rakhmetov@gmail.com; 6 City Multidisciplinary Hospital No. 2, Astana 010000, Kazakhstan; ismailov.almas.490@gmail.com; 7 National Scientific Center of Surgery Named After A.N. Syzganov, Almaty 050000, Kazakhstan; abdykadyrov.m@gmail.com (A.M.); yerlanabdrashev@gmail.com (Y.A.)

**Keywords:** pancreatic cancer, malignant distal biliary obstruction, ERCP, biliary stenting, self-expandable metal stents, recurrent biliary obstruction, endoscopic biliary drainage, complications

## Abstract

**Background/Objectives**: Malignant distal biliary obstruction (MDBO) is a frequent complication of pancreatic cancer and often leads to obstructive jaundice, impaired liver function, and delayed oncologic treatment. Endoscopic biliary drainage using endoscopic retrograde cholangiopancreatography (ERCP) with stent placement is the standard minimally invasive approach for restoring biliary flow. However, clinical outcomes and complication rates vary across studies depending on stent design, placement technique, and patient characteristics. The aim of this systematic review was to evaluate the clinical outcomes and complications associated with endoscopic biliary stenting in pancreatic cancer-related MDBO. **Methods:** A systematic literature search was performed in PubMed/MEDLINE, ScienceDirect, Web of Science, and the Cochrane Library for studies published between January 2016 and January 2026. Studies evaluating ERCP-guided biliary stenting in adult patients with pancreatic cancer-related malignant distal biliary obstruction were included. Study selection followed PRISMA 2020 guidelines, and methodological quality was assessed using the Newcastle–Ottawa Scale. Clinical outcomes including technical success, clinical success, stent patency, recurrent biliary obstruction, and procedure-related complications were analyzed. **Results:** Eighteen studies involving a total of 3291 patients were included in the qualitative synthesis. Technical success rates were consistently high, reaching up to 100% in several studies, while clinical success rates generally exceeded 90%. Median time to recurrent biliary obstruction ranged from approximately 102 to 541 days depending on stent type and placement technique. Recurrent biliary obstruction was the most frequently reported complication, occurring in 30.7% of patients. Stent migration occurred in 14.9% of cases, while post-ERCP pancreatitis was reported in approximately 4.2% of patients. Several studies demonstrated longer patency with self-expandable metal stents compared with plastic stents. **Conclusions:** Endoscopic biliary stenting performed during ERCP is an effective and safe strategy for the management of malignant distal biliary obstruction in pancreatic cancer. Self-expandable metal stents provide more durable biliary drainage and reduce the need for repeat interventions. Nevertheless, recurrent biliary obstruction remains a common limitation, highlighting the need for further improvements in stent technology and optimized placement strategies.

## 1. Introduction

Pancreatic cancer remains one of the most lethal malignant diseases and continues to represent a major global health burden. According to GLOBOCAN 2022 estimates, approximately 495,773 new cases of pancreatic cancer were diagnosed worldwide, with 466,003 deaths attributed to the disease in the same year, reflecting the extremely high mortality associated with this malignancy [1]. Pancreatic cancer currently ranks among the leading causes of cancer-related death globally, and its incidence continues to increase in many regions [2,3]. The prognosis remains poor, with a five-year overall survival rate of only 10–12%, largely due to late diagnosis and aggressive tumor biology [4]. Population-based analyses also indicate that the median overall survival for advanced pancreatic cancer rarely exceeds 6–11 months depending on stage and treatment availability [5].

Tumors located in the head of the pancreas account for approximately 60–70% of pancreatic cancers and, due to their anatomical proximity to the common bile duct, frequently cause malignant distal biliary obstruction (MDBO) [6]. Consequently, obstructive jaundice is one of the most common clinical manifestations of pancreatic head tumors and develops in a substantial proportion of patients during the course of the disease. Observational studies report that up to 70% of patients with pancreatic head carcinoma present with obstructive jaundice at diagnosis [7,8]. In large multicenter cohorts of unresectable pancreatic cancer, most patients require biliary drainage procedures to relieve obstruction and restore bile flow [9].

Persistent biliary obstruction has important clinical consequences. Cholestasis and impaired bile drainage may lead to progressive hepatic dysfunction, bacterial cholangitis, severe pruritus, malabsorption, and deterioration in nutritional status and quality of life [10]. Moreover, untreated biliary obstruction may delay systemic chemotherapy or surgical treatment and is associated with increased morbidity and mortality. Therefore, rapid restoration of biliary drainage is considered a key component in the management of pancreatic cancer-related biliary obstruction [11].

Endoscopic biliary drainage using endoscopic retrograde cholangiopancreatography (ERCP) has become the standard minimally invasive treatment for malignant distal biliary obstruction. The technique enables placement of a biliary stent across the obstructed segment of the bile duct, restoring bile flow and relieving jaundice. ERCP-guided biliary stenting is widely used because it achieves high success rates while avoiding the morbidity associated with surgical bypass or percutaneous drainage procedures [12,13,14,15].

Biliary drainage is usually achieved by placement of either plastic stents (PSs) or self-expandable metal stents (SEMSs). Plastic stents are inexpensive and easy to deploy but have relatively short patency, typically 3–4 months, due to occlusion by biliary sludge or tumor compression [16]. In contrast, SEMSs have a larger luminal diameter and significantly longer patency, often ranging from 6 to 12 months, thereby reducing the need for repeat interventions [17,18]. Several comparative studies have demonstrated that metal stents are associated with lower rates of stent dysfunction and reintervention compared with plastic stents in malignant biliary obstruction [19].

Despite its effectiveness, ERCP-guided biliary stenting is associated with potential complications. Procedure-related adverse events include pancreatitis, cholangitis, bleeding, perforation, and stent dysfunction [20]. Contemporary systematic reviews and large meta-analyses covering over 100,000 procedures indicate an overall ERCP complication rate of approximately 10.2% in the general population [21]. Among specific complications, post-ERCP pancreatitis remains the most common, occurring in approximately 9–10% of cases and reaching higher rates in high-risk patients [22].

Although numerous clinical studies have evaluated endoscopic biliary stenting in malignant biliary obstruction, reported outcomes vary considerably depending on stent type, study design, patient population, and follow-up duration. Differences in reported endpoint-including stent patency, recurrent biliary obstruction, reintervention rates, and complication profiles-make direct comparison of studies difficult. Moreover, many previous studies evaluated heterogeneous populations with different causes of malignant biliary obstruction. As a result, evidence specifically addressing pancreatic cancer-related malignant distal biliary obstruction remains fragmented.

The aim of this systematic review was to analyze and summarize the available evidence regarding clinical outcomes and complications associated with endoscopic biliary stenting in patients with malignant distal biliary obstruction caused by pancreatic cancer. Specifically, the review focuses on technical and clinical success, stent patency, recurrent biliary obstruction, reintervention rates, and procedure-related complications reported in studies published during the last decade.

## 2. Materials and Methods

The study protocol was registered in the PROSPERO International Prospective Register of Systematic Reviews of the National Institute for Health Research (ID: CRD420261343225) [23]. The study was conducted according to the Preferred Reporting Items for Systematic Reviews and Meta-Analyses (PRISMA 2020) recommendations [24]. The completed PRISMA checklist (Table S1) is provided in the Supplementary Materials.

### 2.1. Eligibility Criteria

Eligibility criteria were established prior to the screening process in order to ensure methodological consistency and to avoid selection bias during study inclusion. These criteria were developed based on the clinical objective of the review and the need to ensure comparability of the included studies (Table 1).

The eligibility criteria were formulated to ensure that the included studies reflected contemporary clinical practice and provided comparable clinical data. Restricting the population to adult patients with malignant distal biliary obstruction caused by pancreatic cancer was necessary because the mechanisms of obstruction, therapeutic strategies, and clinical outcomes differ significantly between malignant and benign biliary diseases. Inclusion of benign obstruction could therefore introduce substantial clinical heterogeneity and bias the interpretation of outcomes.

Only studies evaluating endoscopic biliary stenting performed during ERCP were considered eligible because ERCP represents the current standard approach for biliary decompression in pancreatic cancer. Studies investigating surgical bypass or percutaneous drainage alone were excluded since these procedures involve different indications, complication profiles, and outcome measures.

The decision to include randomized trials and observational cohort studies reflects the structure of the available clinical literature in this field. Randomized trials are relatively rare in interventional endoscopy, and many clinically relevant data are derived from well-conducted observational cohorts. However, small case reports and case series involving fewer than ten patients were excluded due to the high risk of publication bias and limited generalizability of their findings.

The time restriction to studies published after 2016 was applied to ensure that the included evidence reflects modern stent technologies, including the widespread clinical adoption of self-expandable metal stents and fully covered stent designs. Earlier studies often evaluated older stent models that are no longer widely used in clinical practice.

Finally, inclusion was limited to studies reporting clinically relevant outcomes such as technical success, stent patency, recurrent biliary obstruction, and procedure-related complications, since these parameters directly influence treatment decisions and patient management.

### 2.2. Search Strategy

A comprehensive electronic search of the literature was performed in four international databases that are widely used for biomedical research: PubMed/MEDLINE, ScienceDirect, Web of Science Core Collection, and the Cochrane Library. These databases were selected because together they provide broad coverage of clinical studies in gastroenterology, oncology, and interventional endoscopy. The search covered publications from 1 January 2016 to 1 January 2026, reflecting the most recent decade of clinical research and the introduction of modern biliary stent technologies.

The search strategy was designed to maximize sensitivity while maintaining adequate specificity for studies evaluating endoscopic biliary drainage in pancreatic cancer. Keywords describing the disease entity, the type of biliary obstruction, and the intervention of interest were combined using Boolean operators AND and OR. Both controlled vocabulary terms (such as MeSH terms in PubMed) and free-text keywords were used to capture studies that might be indexed under different terminology.

The search strategies were adapted to the indexing systems and syntax requirements of each database. Table 2 summarizes the detailed search expressions used in each database together with the number of records identified during the initial search.

**Figure 1:**
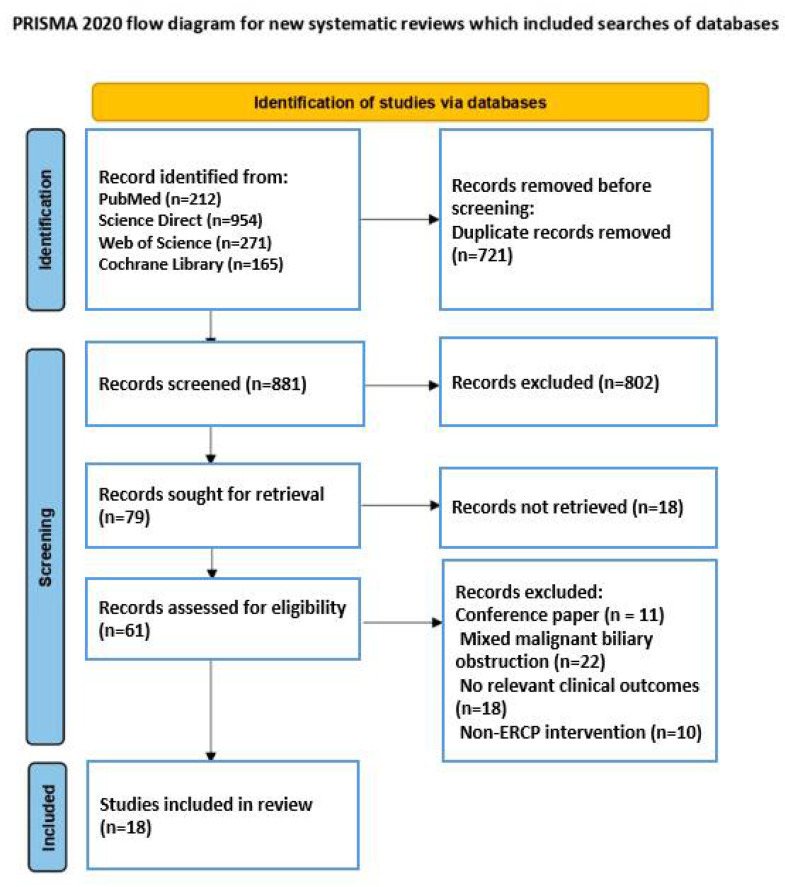
Flow chart of the PRISMA study selection process.

The initial search identified 1602 records across the selected databases. After removal of duplicates, 881 articles remained for screening. Following title and abstract evaluation, 79 studies were assessed in full text, and 18 studies met the eligibility criteria and were included in the qualitative synthesis (Figure 1). Eighteen reports could not be retrieved because only abstract versions were available, and the full texts were not accessible through institutional or online database.

### 2.3. Study Selection and Data Extraction

Study selection was performed independently by two reviewers using a two-stage screening process. Any disagreements between reviewers were resolved by consensus or consultation with a third reviewer. During the first stage, titles and abstracts were reviewed to identify studies potentially relevant to the research question. Articles that appeared to meet the eligibility criteria or for which eligibility could not be determined based on the abstract alone were selected for full-text evaluation. The level of agreement between reviewers during the screening process was high, and disagreements were resolved through discussion and consensus.

During the second stage, the full texts of potentially eligible studies were carefully assessed. Any disagreements regarding study inclusion were resolved through discussion and consensus.

Data extraction was conducted using a standardized extraction form developed prior to the analysis. The extracted variables included author name, year of publication, country of origin, study design, sample size, patient characteristics, type of biliary stent used, duration of follow-up, and reported clinical outcomes.

Particular attention was given to outcomes reflecting the performance and safety of biliary stenting, including technical success of stent placement, clinical success of biliary decompression, duration of stent patency, recurrent biliary obstruction, reintervention rates, and complications such as post-ERCP pancreatitis, cholangitis, bleeding, perforation, and stent migration.

### 2.4. Risk-of-Bias Assessment

The methodological quality of the included studies was evaluated using the Newcastle-Ottawa Scale (NOS), a validated instrument commonly used to assess the risk of bias in observational studies included in systematic reviews. The NOS evaluates studies across three principal domains: the selection of study participants, comparability of study groups, and adequacy of outcome assessment and follow-up.

Each study can receive a maximum score of nine points. Studies scoring 7–9 points were considered to have a low risk of bias, studies scoring 5–6 points were classified as having moderate risk, and studies scoring ≤4 points were considered to have a high risk of bias (Table 3). Quality assessment was performed independently by two reviewers to reduce subjective bias.

Although most studies were rated as having low risk of bias according to the Newcastle–Ottawa Scale, the predominance of retrospective observational designs limits the strength of causal inference. Assessment of publication bias (funnel plot or Egger’s test) was not performed due to the absence of a formal meta-analysis and the substantial heterogeneity among the included studies.

### 2.5. Data Synthesis

A quantitative meta-analysis was not performed because substantial clinical and methodological heterogeneity was observed among the included studies. Differences were identified in several key aspects, including patient populations, tumor stage, stent type (plastic stents, uncovered metal stents, or fully covered metal stents), procedural techniques, follow-up duration, and definitions of clinical outcomes.

Such heterogeneity can introduce significant statistical variability and may lead to misleading pooled estimates if meta-analysis is performed. For this reason, the results of the included studies were synthesized using a qualitative narrative approach, which allowed a comprehensive interpretation of clinical outcomes and complication profiles reported across different study settings. In addition, crude pooled proportions of commonly reported complications were calculated by summing event counts across studies that reported the outcome. These estimates were intended to provide a descriptive overview of complication frequencies and should not be interpreted as formal meta-analytic pooled effects due to substantial clinical and methodological heterogeneity among the included studies.

## 3. Results

### 3.1. Included Study Characteristics

A total of 18 studies were included in the systematic review, comprising an overall cohort of 3291 patients who underwent endoscopic biliary stenting for distal malignant biliary obstruction. When studies included mixed populations with different etiologies of malignant biliary obstruction, pancreatic cancer-specific data were extracted whenever possible. However, in several studies, subgroup-specific data were not available, which may introduce residual heterogeneity in the reported outcomes.

The studies were conducted across several geographic regions. The majority were performed in Japan (n = 13), while additional studies originated from Italy (n = 1), the Netherlands (n = 1), Taiwan (n = 1), South Korea (n = 1), and Romania (n = 1).

With regard to study design, the included studies consisted of 3 randomized controlled trials, 2 prospective multicenter studies, and 13 retrospective cohort studies. Sample sizes varied considerably across studies, ranging from 33 to 1425 patients, reflecting differences in study design and clinical settings.

Most studies included patients with distal malignant biliary obstruction predominantly caused by pancreatic cancer [29,32]. However, several studies also included other etiologies of malignant biliary obstruction, such as cholangiocarcinoma or ampullary tumors [25,26,27,28,31,33,34,35,36,40,41,42].

Across the included studies, various types of self-expandable metal stents (SEMSs) were evaluated, including covered, uncovered, fully covered, and antireflux stents, as well as modifications in stent diameter, design, and placement technique [25,26,27,28,31,34,35,37,40,42].

For the systematic analysis, the following variables were extracted: study design, country, population characteristics, sample size, stent type, and reported clinical outcomes. The main endpoints included time to recurrent biliary obstruction (TRBO), stent patency, and recurrent biliary obstruction (RBO). Additional variables included technical success, clinical success, and procedure-related complications, such as post-ERCP pancreatitis, cholangitis, cholecystitis, and stent migration.

Overall, most studies demonstrated high technical success rates and substantial variability in stent patency outcomes. Reported median TRBO ranged from approximately 102 to 541 days depending on stent type and placement strategy [25,26,27,28,31,33,34,35,36,37,42]. Several comparative studies identified differences in patency or obstruction risk related to stent design or placement technique [25,28,31,34,37].

The detailed characteristics and key findings of the included studies are presented in Table 4.

### 3.2. Clinical Outcomes

Clinical outcomes reported in the included studies mainly addressed stent patency, recurrent biliary obstruction (RBO), and procedure-related complications following endoscopic biliary stenting.

Technical success rates were generally high and reached 100% in several studies [27,28,32,33,35,36,41], while reported clinical success ranged from 90% to 100% [27,28,33,35,36,40,42]. Median time to recurrent biliary obstruction (TRBO) varied across studies, with reported values ranging from approximately 152 to 434 days depending on stent type and placement strategy [27,28,32,33,34,35,36,37,42].

The incidence of recurrent biliary obstruction ranged between 21% and 56% across studies [31,33,35,36,40,41]. Several studies suggested that stent characteristics and placement technique may influence patency outcomes [25,28,31,34,37].

Procedure-related adverse events were reported in multiple studies, including post-ERCP pancreatitis and other complications, with overall rates ranging from 5% to approximately 30% [26,30,33,35,36,38,39,42]. Stent migration was also reported in several cohorts, with incidence ranging from 0% to 36% [26,28,32,33,35,36,40,41]. Detailed clinical outcomes and complications are summarized in Table 5.

Given the substantial clinical and methodological heterogeneity among studies, these values represent crude descriptive proportions calculated from reported event counts rather than pooled meta-analytic estimates. These figures were included to provide an overview of complication frequencies across studies.

Recurrent biliary obstruction was the most frequently reported complication, occurring in 30.7% of patients (347/1130), with reported incidences ranging from 17% to 56% [27,28,31,33,35,36,40]. Stent migration occurred in 14.9% of patients (98/659), with rates varying from 0% to 36% depending on stent design and study population [26,32,33,35,36,40,41]. Post-ERCP pancreatitis was observed in 4.2% of patients (25/589) across studies reporting this outcome [27,30,32,33,38]. Cholangitis and cholecystitis were less frequently reported, with pooled incidences of 4.2% and 6.0%, respectively [32,33,35,36,39]. Detailed distributions of adverse events, including event counts, incidence ranges, and contributing studies, are presented in Table 6. Table 7 summarizes stent patency outcomes and key predictors of recurrent biliary obstruction identified across the included studies.

Median stent patency ranged from 102 to 541 days across the included studies [25,26,27,28,31,32,33,34,35,36,37,42]. Several studies identified predictors of recurrent biliary obstruction (Table 7). Sato et al. reported an increased risk of RBO with covered SEMSs compared with uncovered SEMSs (HR 1.66, 95% CI 1.00–2.76) [31], whereas Hasegawa et al. demonstrated a higher risk of obstruction with laser-cut stents compared with braided stents (OR 2.57, 95% CI 1.045–6.353) [37]. Yamashige et al. found no significant difference in stent patency between 6 mm and 10 mm FCSEMS (HR 1.28, 95% CI 0.67–2.46) [42].

## 4. Discussion

Endoscopic biliary drainage using ERCP represents the standard minimally invasive approach for the management of malignant distal biliary obstruction caused by pancreatic cancer [11,14]. The present systematic review synthesizes evidence from 18 studies published during the past decade and demonstrates that endoscopic biliary stenting provides high procedural success with acceptable complication rates in this patient population [31,33,36]. An important characteristic of the available evidence is the geographic distribution of the included studies. In the present review, 13 of the 18 included studies originated from Japan. Differences in endoscopic practice patterns, healthcare systems, and patient management strategies may influence reported clinical outcomes and may limit the generalizability of these findings to other regions.

The studies included in this review consistently demonstrate that ERCP-guided biliary stenting appears to be a technically reliable and clinically effective intervention for restoring biliary drainage in patients with pancreatic cancer-related obstruction. Several cohorts reported technical success approaching 100% and clinical success exceeding 90% following stent placement [27,28,33,35,36,41]. These findings confirm the central role of ERCP as the primary therapeutic approach for relieving obstructive jaundice in pancreatic cancer.

Despite the high procedural success rates, recurrent biliary obstruction remains an important clinical limitation of endoscopic stenting. Multiple studies included in this review reported substantial rates of recurrent obstruction during follow-up [27,28,31,32,33,35,36,40]. Differences in stent patency across studies likely reflect heterogeneity in tumor stage, stent design, procedural techniques, and follow-up duration. For example, Conio et al. reported longer patency with uncovered self-expandable metal stents compared with covered SEMSs [25], whereas Takada et al. demonstrated improved outcomes with intraductal stent placement compared with transpapillary positioning [28]. Similarly, Ko et al. observed longer stent patency with suprapapillary placement strategies [34].

These findings highlight that both stent architecture and deployment technique may influence long-term biliary drainage outcomes. In addition to tumor progression, mechanisms contributing to stent dysfunction include tumor ingrowth, tumor overgrowth, biliary sludge formation, bacterial biofilm accumulation, and food reflux from the duodenum.

### 4.1. Influence of Stent Design

Several studies included in this review specifically evaluated the impact of stent design on clinical outcomes. Sato et al. demonstrated an increased risk of recurrent biliary obstruction with covered SEMSs compared with uncovered SEMSs [31]. In contrast, Hasegawa et al. reported differences in obstruction rates depending on stent architecture, with laser-cut stents showing inferior patency compared with braided designs [37]. Yamashige et al., however, found no significant difference in patency between different stent diameters when fully covered SEMSs were used [42].

These observations illustrate the complex balance between stent anchoring and tumor ingrowth. Uncovered stents tend to integrate with surrounding tissue, which reduces migration but increases the risk of tumor ingrowth. Covered stents, on the other hand, prevent tumor ingrowth but may be more susceptible to migration. Consequently, the choice of stent should consider both tumor characteristics and expected patient survival.

The findings of the present review are consistent with previous systematic reviews and meta-analyses evaluating biliary stenting for malignant obstruction. Earlier analyses have reported technical success rates exceeding 95% and clinical success rates approaching 90–100% following ERCP-guided biliary drainage [43,44].

Several meta-analyses have also demonstrated that SEMSs provide longer patency and require fewer reinterventions compared with plastic stents in malignant biliary obstruction [45]. This is particularly relevant in pancreatic cancer, where patients with unresectable disease may require prolonged biliary drainage during systemic therapy. In such cases, metal stents reduce the need for repeated ERCP procedures and provide more durable biliary decompression.

### 4.2. Procedure-Related Complications of Endoscopic Biliary Drainage

Although ERCP-guided biliary stenting is considered relatively safe, procedure-related complications remain clinically relevant. Post-ERCP pancreatitis is one of the most common adverse events associated with therapeutic ERCP. Large observational studies report pancreatitis rates typically ranging from approximately 3% to 10%, depending on patient risk factors and procedural techniques [46]. The complication rates reported in the studies included in this review fall within this expected range.

Stent migration represents another clinically significant complication and was reported in several cohorts included in this review [26,32,33,35,36,40,41]. Migration risk is strongly influenced by stent design, with covered SEMSs demonstrating higher migration rates compared with uncovered stents. Conversely, uncovered stents may become obstructed due to tumor ingrowth over time [47]. Careful selection of stent type is therefore important in order to balance these competing risks.

Although the present review focuses on endoscopic biliary stenting, several studies in the broader pancreatic cancer literature have explored additional biological and tumor-related predictors of postoperative outcomes, including inflammatory hematological markers and perineural tumor infiltration [48,49,50,51].

Other complications such as cholangitis and cholecystitis may occur when biliary drainage is incomplete or when the cystic duct becomes obstructed by the stent. Prompt recognition and management of these complications are essential for maintaining clinical stability in patients with advanced malignancy.

In recent years, endoscopic ultrasound-guided biliary drainage (EUS-BD) has emerged as an alternative endoscopic approach for the management of malignant distal biliary obstruction, particularly in cases where ERCP is unsuccessful or technically challenging. Recent randomized trials and systematic reviews suggest that EUS-BD provides technical and clinical success rates comparable to ERCP while potentially reducing the risk of post-procedure pancreatitis and the need for repeat interventions [44,52,53,54]. These findings highlight the evolving role of EUS-guided techniques in the management of biliary obstruction and underscore the importance of considering EUS-BD within the broader context of contemporary endoscopic drainage strategies.

### 4.3. Contemporary Evidence and Evolving Stent Strategies

Recent research continues to refine the optimal approach to biliary stenting in malignant obstruction. Contemporary cohort studies confirm that SEMS placement is associated with improved stent patency and fewer repeat interventions compared with plastic stents in patients with pancreatic cancer-related biliary obstruction [55]. In addition, recent systematic reviews have demonstrated that metal stents are more cost-effective than plastic stents when longer expected survival requires durable biliary drainage [56].

Importantly, the present review highlights that recurrent biliary obstruction remains the most frequently reported complication following endoscopic biliary stenting in pancreatic cancer-related MDBO, occurring in approximately one third of patients across the analyzed studies. This finding underscores the clinical challenge of maintaining durable biliary drainage in patients with progressive pancreatic malignancy. Despite advances in stent technology, the high incidence of RBO suggests that current stent designs may still be limited by tumor progression, biofilm formation, and duodenobiliary reflux. These observations emphasize the need for further improvements in stent engineering and individualized stent selection strategies.

Technological developments in stent design have also aimed to reduce obstruction and migration. Anti-reflux metal stents and novel fully covered stent systems have been evaluated in recent prospective studies and may reduce duodenobiliary reflux and prolong stent patency [57]. Furthermore, new stent architectures incorporating anchoring mechanisms and modified mesh structures have been developed in an effort to reduce migration while maintaining resistance to tumor ingrowth [58].

Optimization of stent positioning strategies has also been explored. Several recent studies suggest that suprapapillary stent placement may reduce duodenobiliary reflux and prolong stent function in selected patients with distal malignant obstruction [59]. However, evidence remains heterogeneous and further randomized studies are required to clarify the clinical benefit of these approaches.

### 4.4. Guideline Recommendations and Clinical Implications

The results of this review are consistent with current international guideline recommendations. The European Society of Gastrointestinal Endoscopy (ESGE) recommends ERCP-guided biliary drainage using self-expandable metal stents as the preferred approach for palliation of distal malignant biliary obstruction, particularly in patients with unresectable pancreatic cancer [60]. These recommendations are based on evidence demonstrating superior stent patency and reduced need for reintervention compared with plastic stents.

Similarly, the American Society for Gastrointestinal Endoscopy (ASGE) guidelines recommend metal stent placement for patients with malignant biliary obstruction who are not candidates for immediate surgical resection [61]. Metal stents are preferred due to their longer functional lifespan and lower procedural burden.

Oncological guidelines also highlight the importance of effective biliary decompression in pancreatic cancer. The National Comprehensive Cancer Network (NCCN) recommends endoscopic biliary drainage in patients with obstructive jaundice prior to initiating systemic therapy, as persistent cholestasis may delay chemotherapy and increase the risk of infectious complications [62]. Adequate biliary drainage therefore represents a critical component of multidisciplinary management of pancreatic cancer.

The findings of the present systematic review support these guideline recommendations. The consistently high technical success rates observed across studies, together with the longer patency reported for metal stents, support the current preference for SEMSs in patients with unresectable pancreatic cancer and malignant distal biliary obstruction. At the same time, the relatively high incidence of recurrent biliary obstruction observed in the analyzed studies suggests that even modern SEMSs do not completely eliminate the risk of stent dysfunction. Therefore, continued refinement of stent technology and optimization of placement strategies remain essential to further improve long-term outcomes.

To our knowledge, this review provides one of the most comprehensive recent summaries of clinical outcomes and complication profiles associated with endoscopic biliary stenting specifically in pancreatic cancer-related malignant distal biliary obstruction.

Another important observation from this review concerns the structure of the available evidence. Most studies included in the analysis were retrospective observational cohorts, while randomized controlled trials remain relatively limited. Future research should prioritize well-designed prospective multicenter studies and randomized trials comparing different stent designs, placement techniques, and anti-reflux technologies. Standardized definitions of outcomes such as recurrent biliary obstruction, stent dysfunction, and procedure-related complications would also improve comparability across studies. Therefore, the certainty of the available evidence should be interpreted with caution due to the predominance of retrospective observational studies and the heterogeneity of the included cohorts.

### 4.5. Limitations

Several limitations should be considered when interpreting the findings of this systematic review.

First, substantial clinical and methodological heterogeneity was observed across the included studies. The analyzed cohorts differed in several important aspects, including study design, patient characteristics, tumor stage, stent type, stent diameter, placement technique, and follow-up duration. In addition, definitions of key clinical outcomes such as recurrent biliary obstruction, stent dysfunction, and adverse events were not entirely uniform across studies. Such variability may limit direct comparison of results and complicate interpretation of overall reported estimates of stent patency and complication rates.

Second, the majority of studies included in this review were retrospective observational cohorts. Although these studies provide valuable real-world data, they are inherently susceptible to selection bias, confounding factors, and incomplete reporting of outcomes. Randomized controlled trials in this field remain relatively limited, and therefore the overall strength of evidence is influenced by the predominance of observational data.

Third, follow-up duration varied considerably across studies and was often influenced by differences in patient survival. Since pancreatic cancer is associated with a poor prognosis, many patients experience limited follow-up periods, which may lead to underestimation of late complications such as stent occlusion, migration, or recurrent biliary obstruction. Conversely, studies involving patients with longer survival may report higher cumulative rates of stent dysfunction simply due to extended observation periods.

Fourth, although the present review focused on pancreatic cancer-related MDBO, several included studies enrolled mixed populations with different causes of malignant biliary obstruction, including cholangiocarcinoma or ampullary tumors. Although pancreatic cancer-specific data were extracted whenever available, subgroup-specific data were not consistently reported across all studies, which may introduce residual heterogeneity in the reported outcomes.

Fifth, variability in stent technology and procedural strategies across studies should also be considered. Over the past decade, significant advances have occurred in stent design, including improvements in covering materials, anti-migration features, and anti-reflux mechanisms. Earlier studies included in the review may therefore reflect older stent technologies that are not fully representative of current clinical practice.

Sixth, another limitation relates to the literature search strategy. Although several major databases were included in the search, EMBASE was not specifically searched. As EMBASE is an important biomedical database that indexes a large number of gastroenterology and oncology studies, it is possible that some relevant publications may not have been captured in the present review.

Finally, although a comprehensive literature search was performed across multiple databases, the possibility of publication bias cannot be completely excluded. Studies with favorable procedural outcomes are more likely to be published, whereas smaller studies with negative or inconclusive results may remain unpublished.

Despite these limitations, the present review provides a comprehensive overview of contemporary evidence on endoscopic biliary stenting in pancreatic cancer-related MDBO and highlights important clinical patterns across recent studies.

## 5. Conclusions

Endoscopic biliary stenting performed during ERCP remains a widely used and appears to be an effective strategy for the management of malignant distal biliary obstruction in patients with pancreatic cancer. The evidence synthesized in this systematic review indicates consistently high technical and clinical success rates across the included studies, supporting the central role of ERCP-guided biliary drainage in the palliative management of obstructive jaundice in this population.

Self-expandable metal stents appear to provide more durable biliary decompression and may reduce the need for repeated endoscopic interventions compared with plastic stents. However, recurrent biliary obstruction remains a frequent clinical challenge, reflecting both tumor progression and mechanical limitations of currently available stent technologies.

Procedure-related complications such as pancreatitis, cholangitis, and stent migration occur in a minority of patients but remain clinically important and require careful monitoring following ERCP. Selection of stent type and placement strategy should therefore consider tumor characteristics, expected patient survival, and the anticipated duration of biliary drainage.

Future research should focus on improving stent design, optimizing placement strategies, and identifying patient-specific predictors of stent dysfunction. Well-designed prospective studies and randomized trials will be important for further clarifying the most effective stent technologies and procedural approaches for achieving durable biliary drainage in patients with pancreatic cancer-related biliary obstruction.

## Figures and Tables

**Table 1:** Eligibility criteria for study selection.

Category	Inclusion Criteria	Exclusion Criteria
Population	Adult patients with malignant distal biliary obstruction caused by pancreatic cancer	Benign biliary obstruction or non-malignant etiologies
Intervention	Endoscopic biliary stenting performed during ERCP	Surgical biliary bypass or percutaneous drainage without endoscopic intervention
Study design	Randomized controlled trials, prospective cohort studies, retrospective cohort studies, multicenter clinical studies	Case reports, case series with fewer than 10 patients, narrative reviews, editorials
Outcomes	Studies reporting at least one relevant clinical outcome (technical success, stent patency, complications, or survival)	Studies without clinical outcome data
Publication period	January 2016–January 2026	Studies published before 2016
Language	English	Non-English publications without accessible translation

**Table 2:** Detailed literature search strategy and number of identified records.

Database	Search Strategy	Records Identified
PubMed/MEDLINE	(“Pancreatic Neoplasms” [Mesh] OR “pancreatic cancer” [Title/Abstract] OR “pancreatic adenocarcinoma” [Title/Abstract]) AND (“Biliary Tract Obstruction” [Mesh] OR “biliary obstruction” [Title/Abstract] OR “malignant biliary obstruction” [Title/Abstract]) AND (“ERCP” [Title/Abstract] OR “endoscopic retrograde cholangiopancreatography” [Title/Abstract] OR “biliary stent” [Title/Abstract] OR “biliary drainage” [Title/Abstract])	212
ScienceDirect	(“pancreatic cancer” OR “pancreatic adenocarcinoma”) AND (“malignant biliary obstruction” OR “distal biliary obstruction”) AND (“ERCP” OR “endoscopic biliary stent” OR “biliary drainage”)	954
Web of Science	TS = (“pancreatic cancer” OR “pancreatic adenocarcinoma”) AND TS = (“malignant biliary obstruction” OR “distal biliary obstruction”) AND TS = (“ERCP” OR “biliary stent” OR “endoscopic biliary drainage”)	271
Cochrane Library	(“pancreatic cancer”) AND (“biliary stent” OR “biliary drainage” OR “ERCP”)	165

**Table 3:** Risk-of-bias assessment using the Newcastle–Ottawa Scale (NOS).

Study	Selection (0–4)	Comparability (0–2)	Outcome (0–3)	Total Score	Risk of Bias
Conio M et al. [25]	4	2	3	9	Low
Hamada T et al. [26]	4	2	3	9	Low
Inoue T et al. [27]	3	1	3	7	Low
Takada T et al. [28]	3	1	3	7	Low
Constantinescu A et al. [29]	3	1	2	6	Moderate
Harada R et al. [30]	3	1	2	6	Moderate
Sato T et al. [31]	4	2	3	9	Low
Wu CH et al. [32]	4	2	3	9	Low
Yamada M et al. [33]	3	1	3	7	Low
Ko SW et al. [34]	3	1	3	7	Low
Nakagawa H et al. [35]	3	1	3	7	Low
Sakai A et al. [36]	3	1	3	7	Low
Hasegawa S et al. [37]	4	2	3	9	Low
Onnekink AM et al. [38]	4	2	3	9	Low
Tamura T et al. [39]	4	2	3	9	Low
Kohashi S et al. [40]	3	1	3	7	Low
Takahashi S et al. [41]	3	1	3	7	Low
Yamashige D et al. [42]	4	2	3	9	Low

**Table 4:** General Characteristics of Included Studies.

Author	Year	Country	Study Design	Population	n	Intervention/Stent Type	Primary Outcome	Key Results
Conio M et al. [25]	2018	Italy	Randomized controlled trial	Distal malignant biliary obstruction	158	Covered vs. uncovered SEMS	TRBO	Median TRBO: 240 vs. 541 days; *p* = 0.031
Hamada T et al. [26]	2019	Japan	Multicenter RCT	Unresectable distal malignant biliary obstruction	104	Antireflux SEMS vs. covered SEMS	Time to recurrent biliary obstruction (TRBO)	Median TRBO: 251 vs. 351 days; *p* = 0.11; migration: 31% vs. 12%
Inoue T et al. [27]	2020	Japan	Prospective multicenter	Distal malignant biliary obstruction	40	Intraductal FCSEMS	TRBO	Technical success 100%; clinical success 95%; median TRBO 339 days
Takada T et al. [28]	2020	Japan	Retrospective cohort	Distal malignant biliary obstruction	73	Intraductal vs. transpapillary SEMS	TRBO	Median TRBO: 307 vs. 161 days; *p* = 0.022
Constantinescu A et al. [29]	2020	Romania	Retrospective cohort	Pancreatic cancer with biliary obstruction	115	Plastic vs. metal stent	Clinical outcomes	30-day mortality 5%; fewer ERCP procedures with metal stents
Harada R et al. [30]	2020	Japan	Retrospective cohort	Distal malignant biliary obstruction	33	SEMS with pancreatic stent	Post-ERCP pancreatitis	PEP reduced vs. control
Sato T et al. [31]	2022	Japan	Multicenter cohort	Distal malignant biliary obstruction	238	14 mm uncovered SEMS vs. 10 mm covered SEMS	RBO/TRBO	RBO: 25% vs. 37%; HR 1.66 (95% CI 1.00–2.76); *p* = 0.04
Wu CH et al. [32]	2023	Taiwan	Retrospective cohort	PDAC with distal malignant biliary obstruction	159	FCSEMS primary vs. FCSEMS after plastic stent	Recurrent biliary obstruction (RBO)	RBO: 22 vs. 18 cases; no difference in stent patency; stent length > 6 cm risk factor for RBO
Yamada M et al. [33]	2023	Japan	Retrospective cohort	Distal malignant biliary obstruction	86	Covered SEMS	RBO/patency	TRBO: 347 vs. 294 days; *p* = 0.189
Ko SW et al. [34]	2024	Korea	Comparative cohort	Distal malignant biliary obstruction	84	Suprapapillary vs. transpapillary SEMS	Stent patency	Median patency: 369 vs. 154 days; *p* < 0.01
Nakagawa H et al. [35]	2024	Japan	Retrospective cohort	Distal malignant biliary obstruction	40	PTFE-covered SEMS	TRBO/RBO	Median TRBO: 434 vs. 102 days; *p* = 0.10
Sakai A et al. [36]	2024	Japan	Prospective multicenter	Distal malignant biliary obstruction	73	Novel FCSEMS	TRBO	Median TRBO ~300 days
Hasegawa S et al. [37]	2024	Japan	Randomized controlled trial	Unresectable distal malignant biliary obstruction	56	Braided vs. laser-cut FCSEMS	RBO	OR 2.57 (95% CI 1.045–6.353); TRBO 418 vs. 220 days; *p* = 0.0118
Onnekink AM et al. [38]	2024	Netherlands	Multicenter RCT	Suspected distal malignant biliary obstruction	297	Sphincterotomy vs. no sphincterotomy before FCSEMS	Post-ERCP pancreatitis	PEP: 17% vs. 21% RR 0.78 (95% CI 0.49–1.26); *p* = 0.37
Tamura T et al. [39]	2024	Japan	Multicenter retrospective study	Distal malignant biliary obstruction	1425	Self-expandable metal stent placement	Adverse events	Early adverse events 16.0%; recurrent biliary obstruction 27.6%
Kohashi S et al. [40]	2025	Japan	Multicenter cohort	Distal malignant biliary obstruction	81	PCSEMS vs. FCSEMS	RBO	RBO: 21% vs. 43%; *p* = 0.036; migration: 3% vs. 36%; *p* < 0.001
Takahashi S et al. [41]	2025	Japan	Multicenter cohort	Distal malignant biliary obstruction	111	Multi-hole FCSEMS	RBO	Technical success 100%; RBO 21%; migration 1.9%
Yamashige D et al. [42]	2025	Japan	Propensity-matched cohort	Distal malignant biliary obstruction	118	FCSEMS 6 mm vs. 10 mm	TRBO	Median TRBO: 287 vs. 286 days; *p* = 0.458

**Table 5:** Clinical outcomes and complications of endoscopic biliary stenting in pancreatic cancer-related MDBO.

Author	n	Technical Success	Clinical Success	TRBO/Stent Patency	RBO	Adverse Events	Migration
Conio M et al. [25]	158	NR	NR	240 vs. 541 days	NR	NR	NR
Hamada T et al. [26]	104	NR	NR	251 vs. 351 days	NR	20% vs. 18%	31% vs. 12%
Inoue T et al. [27]	40	100%	95%	median 339 days	33%	5%	0%
Takada T et al. [28]	73	100%	100% vs. 98%	307 vs. 161 days	43% vs. 52%	7%	0% vs. 16%
Constantinescu A et al. [29]	115	procedure success reported	NR	NR	NR	NR	NR
Harada R et al. [30]	33	NR	NR	NR	NR	PEP 5% vs. 31%	NR
Sato T et al. [31]	238	NR	NR	not reached vs. 290 days	25% vs. 37%	NR	NR
Wu CH et al. [32]	159	100%	NR	152 vs. 177.5 days	22 vs. 18 cases	pancreatitis 2.9–3.6%	migration 11–13%
Yamada M et al. [33]	86	100%	95% vs. 100%	347 vs. 294 days	24% vs. 44%	11% vs. 23%	3% vs. 10%
Ko SW et al. [34]	84	NR	NR	369 vs. 154 days	NR	NR	NR
Nakagawa H et al. [35]	40	100%	90%	434 vs. 102 days	56% vs. 50%	15–20%	39% vs. 11%
Sakai A et al. [36]	73	100%	97.3%	median 216 days	49.3%	20.5%	35.6%
Hasegawa S et al. [37]	56	NR	NR	418 vs. 220 days	17.2% vs. 44.4%	NR	NR
Onnekink AM et al. [38]	297	NR	NR	NR	NR	PEP 17% vs. 21%	NR
Tamura T et al. [39]	1425	NR	NR	NR	27.6%	early adverse events 16%	NR
Kohashi S et al. [40]	81	NR	100% vs. 98%	NR	21% vs. 43%	21% vs. 19%	3% vs. 36%
Takahashi S et al. [41]	111	100%	NR	NR	21%	NR	1.9%
Yamashige D et al. [42]	118	NR	94.7% vs. 95.6%	287 vs. 286 days	NR	12% vs. 30.9%	NR

Note: Definitions of key outcomes such as clinical success, recurrent biliary obstruction, and adverse events varied across studies. A summary of outcome definitions used in the included studies is provided in Supplementary Table S2.

**Table 6:** Incidence of adverse events after endoscopic biliary stenting.

Outcome/Complication	Patients with Event (n/N)	Incidence (%)	Range Across Studies	Studies
Post-ERCP pancreatitis	25/589	4.2%	2.5–31%	[27,30,32,33,38]
Cholangitis	31/731	4.2%	0–15%	[33,35,36,39]
Cholecystitis	19/318	6.0%	3–13%	[32,33,36]
Stent migration	98/659	14.9%	0–36%	[26,32,33,35,36,40,41]
Stent occlusion	113/563	20.1%	17–29%	[31,32,35,36,39]
Sludge/food impaction	12/104	11.5%	9.8–13%	[26]
Tumor ingrowth	12/69	17.4%	up to 18%	[39]
Tumor overgrowth	6/199	3.0%	4–6%	[27,32]
Non-occlusion cholangitis	10/69	14.5%	up to 15%	[39]
Recurrent biliary obstruction (RBO)	347/1130	30.7%	17–56%	[27,28,31,32,33,35,36,40]
Early adverse events	246/1504	16.4%	16–23%	[33,36,39]
Overall adverse events	92/356	25.8%	12–30.9%	[33,36,42]
Procedure-related mortality (30-day)	6/115	5.2%	5%	[29]

Note: Incidence values were calculated using the number of patients from studies that reported the specific complication outcome. Therefore, denominators differ across outcomes.

**Table 7:** Stent patency and predictors of recurrent biliary obstruction.

Outcome	Reported Value	Studies
Median stent patency (TRBO)	102–541 days	[25,26,27,28,31,32,33,34,35,36,37,42]
Median overall survival	158–233 days	[31,36,37]
Longer patency with uncovered SEMS	HR 1.66 (95% CI 1.00–2.76)	[31]
Risk of RBO depending on stent design	OR 2.57 (95% CI 1.045–6.353)	[37]
No difference in patency between 6 mm and 10 mm FCSEMS	HR 1.28 (95% CI 0.67–2.46)	[42]
Reduced risk of migration with PCSEMS	sHR 0.077 (95% CI 0.01–0.60)	[40]
Risk factor for RBO (stent length > 6 cm)	HR 0.631 (95% CI 0.414–0.691)	[32]

## Data Availability

The original contributions presented in this study are included in the article/Supplementary Material. Further inquiries can be directed to the corresponding author.

## Supplementary Materials

The following supporting information can be downloaded at: https://www.mdpi.com/article/10.3390/jcm15083126/s1. Table S1: The PRISMA checklist; Table S2: Definitions of key clinical outcomes across included studies.

## Abbreviations

ASGE	American Society for Gastrointestinal Endoscopy
CI	Confidence Interval
ERCP	Endoscopic Retrograde Cholangiopancreatography
ESGE	European Society of Gastrointestinal Endoscopy
EUS-BD	Endoscopic Ultrasound-Guided Biliary Drainage
FCSEMS	Fully Covered Self-Expandable Metal Stent
HR	Hazard Ratio
MDBO	Malignant Distal Biliary Obstruction
NCCN	National Comprehensive Cancer Network
NOS	Newcastle–Ottawa Scale
OR	Odds Ratio
PEP	Post-ERCP Pancreatitis
PRISMA	Preferred Reporting Items for Systematic Reviews and Meta-Analyses
PS	Plastic Stent
RBO	Recurrent Biliary Obstruction
RCT	Randomized Controlled Trial
SEMS	Self-Expandable Metal Stent
TRBO	Time to Recurrent Biliary Obstruction
